# Ubiquitin-like 3 as a new protein-sorting factor for small extracellular vesicles

**DOI:** 10.1247/csf.21078

**Published:** 2022-02-23

**Authors:** Yusuke Takanashi, Tomoaki Kahyo, Sae Kamamoto, Hengsen Zhang, Bin Chen, Yashuang Ping, Kiyomichi Mizuno, Akikazu Kawase, Kei Koizumi, Masanori Satou, Kazuhito Funai, Norihiko Shiiya, Mitsutoshi Setou

**Affiliations:** 1 Department of Cellular and Molecular Anatomy, Hamamatsu University School of Medicine, 1-20-1 Handayama, Higashi Ward, Hamamatsu, Shizuoka 431-3192, Japan; 2 First Department of Surgery, Hamamatsu University School of Medicine, 1-20-1 Handayama, Higashi Ward, Hamamatsu, Shizuoka 431-3192, Japan; 3 International Mass Imaging Center, Hamamatsu University School of Medicine, 1-20-1 Handayama, Higashi Ward, Hamamatsu, Shizuoka 431-3192, Japan; 4 Department of Systems Molecular Anatomy, Institute for Medical Photonics Research, Hamamatsu University School of Medicine, 1-20-1 Handayama, Higashi Ward, Hamamatsu, Shizuoka 431-3192, Japan

**Keywords:** UBL3, small extracellular vesicles, protein sorting, ubiquitin-like protein, post-translational modification

## Abstract

Ubiquitin-like 3 (UBL3) is a well-conserved ubiquitin-like protein (UBL) in eukaryotes and regulates the ubiquitin cascade, but the significant roles of UBL3 in cellular processes remained unknown. Recently, UBL3 was elucidated to be a post-translational modification factor that promotes protein sorting to small extracellular vesicles (sEVs). Proteins sorted into sEVs have been studied as etiologies of sEV-related diseases. Also, there have been attempts to construct drug delivery systems (DDSs) by loading proteins into sEVs. In this review, we introduce the new concept that UBL3 has a critical role in the protein-sorting system and compare structure conservation between UBL3 and other UBLs from an evolutionary perspective. We conclude with future perspectives for the utility of UBL3 in sEV-related diseases and DDS.

## Introduction

Ubiquitin (Ub) and ubiquitin-like proteins (UBLs) control the localization and stability of proteins via post-translational modification (PTM). They promote cellular physiological processes, including protein degradation and transcription, autophagy, endocytosis, the cell cycle, signal transduction and DNA repair. The specific roles of Ub and various UBL modifier systems in cellular physiological processes have been clarified (Tanaka *et al.*, 1998; Vierstra, 2012). For example, the Ub-proteasome system involved in protein degradation (Tanaka *et al.*, 1998), and the autophagy-related protein (ATG)8 and ATG12 systems that promote autophagy (Mizushima, 2020), are prominent proteinous PTM systems. In the Ub-proteasome system, Ub is conjugated covalently to substrate proteins and sorts them into proteasomes. In the ATG8 and ATG12 autophagy system, ATG8 conjugate covalently to phosphatidylethanolamine to form autophagosomes to which substrate proteins are sorted. Ubiquitin-like 3 (UBL3) is a well-conserved UBL in eukaryotes and regulates the Ub-cascade (Dowil *et al.*, 2011; Downes *et al.*, 2006; Lu *et al.*, 2016). However, significant physiological roles of UBL3 in cellular processes remained obscure.

**Fig. 1:**
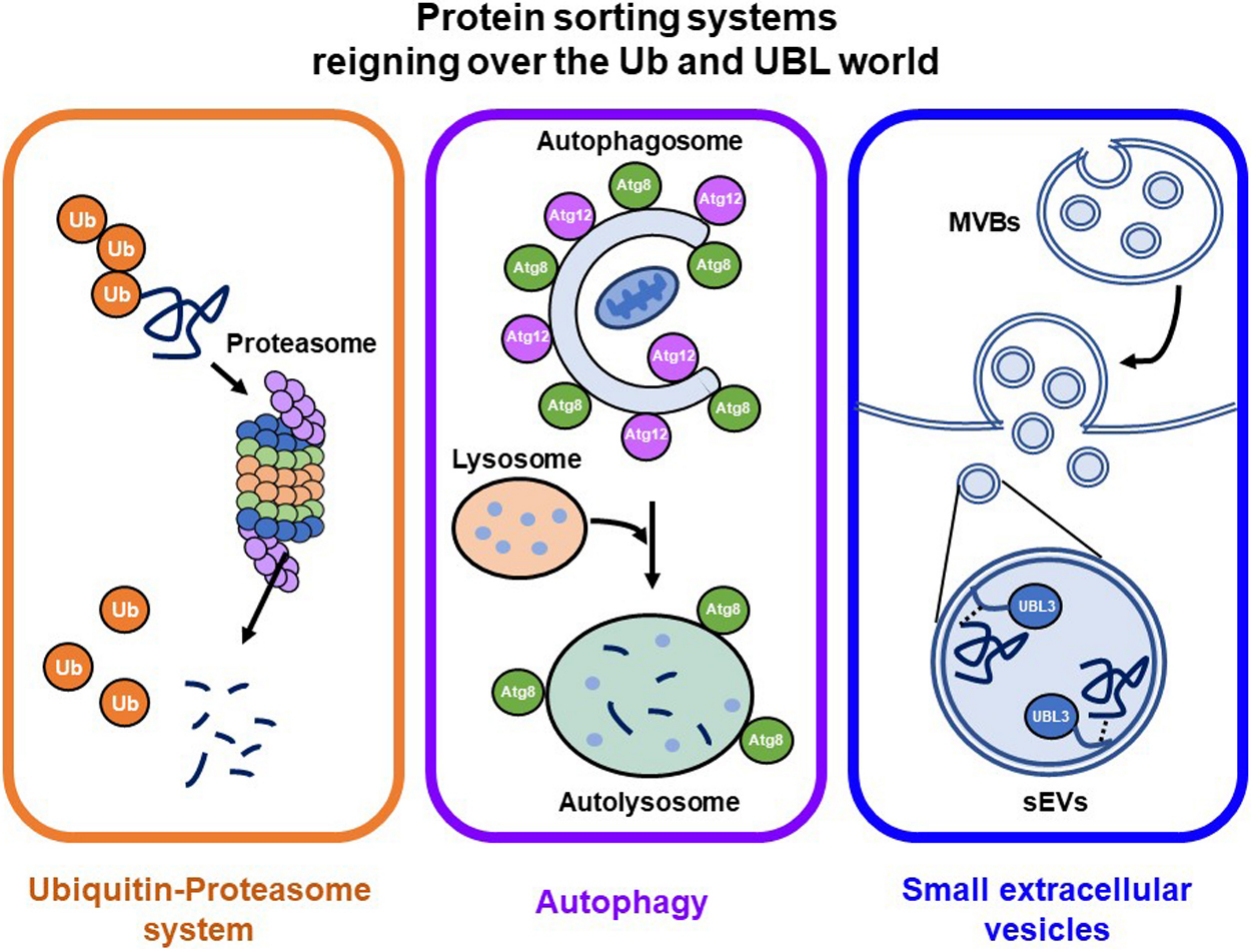
UBL3 as a new protein-sorting factor, joining Ub and UBL The new concept of UBL3 that participate in protein sorting to sEVs, ranking UBL3 alongside the prominent protein-sorting systems, Ub-proteasome and autophagy systems. ATG, autophagy-related protein; MVBs, multivesicular bodies; sEVs, small extracellular vesicles; Ub, ubiquitin; UBL, ubiquitin-like protein; UBL3, ubiquitin-like 3.

Recently, UBL3 was shown to be a PTM factor that contributes to protein sorting to small extracellular vesicles (sEVs) (Ageta *et al.*, 2018; Ageta and Tsuchida, 2019). sEVs are membrane vesicles around 100 nm in diameter secreted from the luminal membranes of multivesicular bodies (MVBs) of various cell types. sEVs contain functional biomolecules, including proteins, lipids, RNA and DNA, that can be horizontally transferred to recipient cells to promote cell-to-cell communication (Simons and Raposo, 2009). Research on sEV-related diseases (cancer and neurodegenerative diseases) and sEV applications as drug delivery systems (DDSs) and diagnostic biomarkers have recently increased dramatically (Antimisiaris *et al.*, 2018; Fujita *et al.*, 2016; Johnsen *et al.*, 2014; Vinaiphat and Sze, 2019). The molecular mechanisms by which proteins are sorted to sEVs are still not fully elucidated, and thus this new concept of UBL3 that contributes to protein sorting to sEVs was considered a significant advancement. UBL3 now joins the prominent protein-sorting systems, the Ub-proteasome and ATG8/ATG12 autophagy systems (Fig. 1). Given the significant physiological role of UBL3 on cellular processes and its implied involvement in sEV-related diseases, UBL3 is likely a key molecule for regulating sEVs.

In this review, we introduce the concept that UBL3 contributes to protein sorting via PTM and compare UBL3 and other UBL family members from the viewpoint of their evolutionarily conserved characteristic structures. We also discuss future perspectives for UBL3 research based on recent research on sEV-related diseases and sEV applications for DDS.

## Ub and UBLs

Ub is a 76-amino acid protein that is highly conserved in eukaryotes and acts as a protein modifier covalently attached to many substrate proteins. Covalent bonding between Ub and a substrate protein is mediated by a three-enzyme cascade (E1: Ub-activating enzyme, E2: Ub-conjugating enzyme, and E3: Ub-ligase) that activates Ub and selects specific substrate proteins (Ciechanover *et al.*, 1984; Finley *et al.*, 1984; Hochstrasser, 2009; Tanaka *et al.*, 1998). This PTM of substrate proteins targets them for degradation by sorting them to a large multi-subunit protease called the 26S proteasome system. The roles of Ub in cellular processes via protein PTM are diverse, such as transcriptional regulation, endocytosis, the cell cycle, signal transduction, and DNA repair. Ub thus represents a breakthrough in understanding protein PTM systems (Tanaka *et al.*, 1998; Vierstra, 2012).

The discovery of diverse proteins that harbor Ub-like structures, called UBLs, increased interest in this protein. Several UBLs act as protein PTM factors in a fashion similar to Ub, and include small ubiquitin-like modifiers (SUMOs); neuronal precursor cell-expressed, developmentally down-regulated 8 (NEDD8); ATG8; ATG12; and ubiquitin-fold modifier 1 (UFM1). These UBLs modify unique substrate proteins or lipids to play discrete roles in cellular processes, ranging from cellular housekeeping, autophagic nutrient recycling, transcriptional regulation, chromatin remodeling, and RNA metabolism (Ageta and Tsuchida, 2019; Vierstra, 2012). However, the biological roles of some UBLs remain obscure, although the role of UBL3, a well-conserved UBL in eukaryotes except for yeasts, was recently elucidated.

## Identification of UBL3

The *UBL3* gene was first identified in *Drosophila melanogaster* (Dm) by Chadwick *et al.* in 1999 (Chadwick *et al.*, 1999). By searching an expressed sequence tag database using the Dm*UBL3* cDNA as a query, they isolated *Homo sapiens* (Hs) and *Mus musculus* (Mm)*UBL3* from keratinocyte stem cells and the testis, respectively. The Hs and Mm*UBL3* mapping confirmed that they localize at the chromosome band 13q12-q13 and the telomeric end of chromosome 5, band G2-3. As UBL3 orthologs were identified in several eukaryotes but not in yeasts by a BLAST search, UBL3 was considered to present only in multi cellular organisms. Besides, although UBL3 was demonstrated to express in various human tissues, its role was unknown.

## Comparison of the structure of UBL3 with Ub and other UBLs

**Fig. 2:**
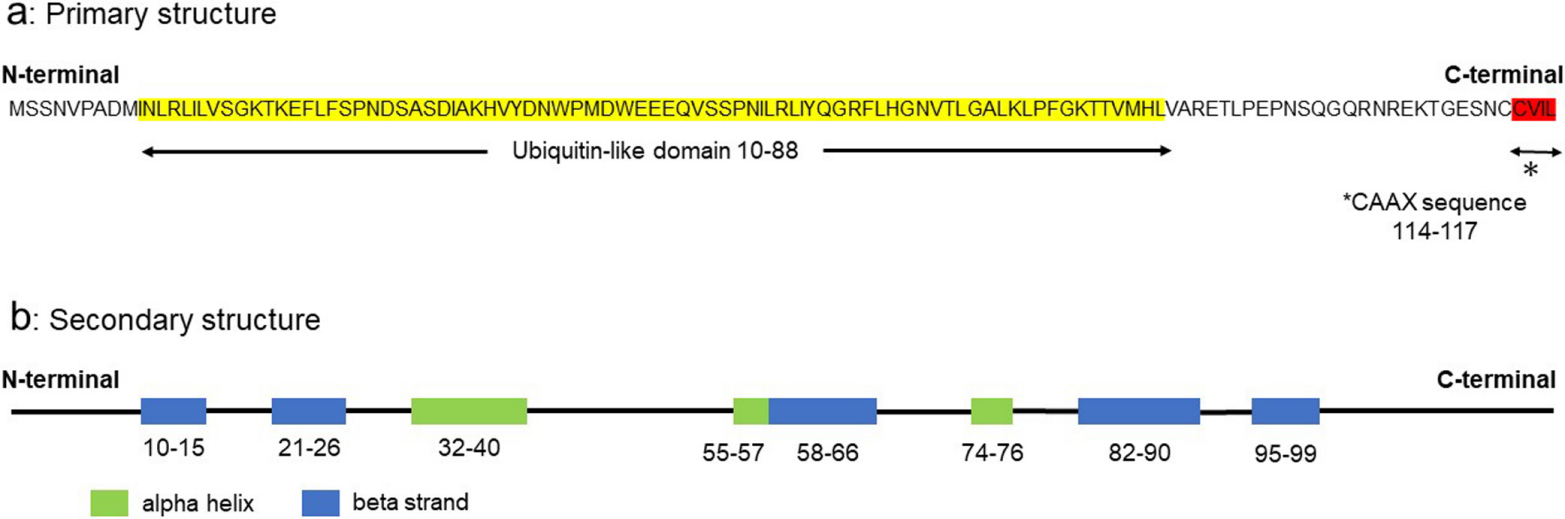
The primary and secondary structures of human UBL3 The characteristics of the primary structure of UBL3 are a ubiquitin-like domain from residues 10 to 88 (yellow) and a CAAX sequence (CVIL) at the end of the carboxyl-terminal (red). The secondary structure elements (α-helix: green, β-sheet: blue) are arranged in a ββαβββ order. UBL3, ubiquitin-like 3.

**Fig. 3:**
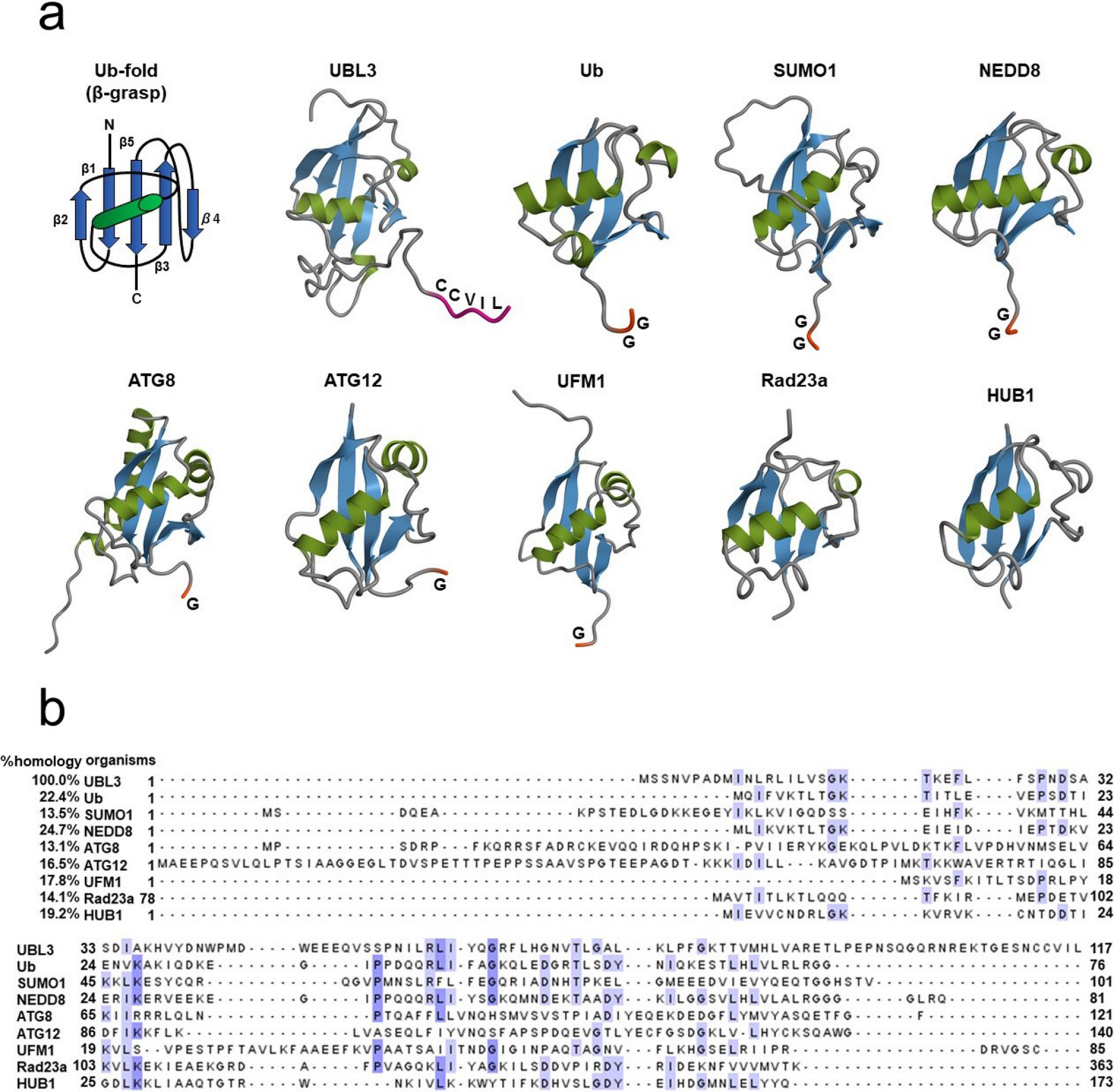
Comparison of the tertiary structures and amino acid sequences of UBL3, Ub and other UBLs (a) The tertiary structure of the Ub-fold (β-grasp) is shared in ubiquitin and other UBLs, including UBL3. Carboxyl-termini of Ub and the majority of UBLs end in glycine residues, except for UBL3, Rad23a and HUB1. UBL3 is distinguished from other UBLs by a CAAX sequence located at its carboxyl-terminal. As for Rad23a, only the Ub-fold, not the full-length, is described. Three-dimensional diagrams of the Ub/UBLs were drawn using QueMol 2.0 (http://www.cuemol.org/ja/) and the following Protein Data Bank IDs (http://www.rcsb.org/): human UBL3, 2GOW; human Ub, 1C3T: human SUMO-1, 2N1V: human NEDD8, 2KO3: humanATG8, 4ZDV: human ATG12, 4GDL: human UFM1, 1WXS: human Rad23a, 1P98: human HUB1, 1POR. (b) Multiple sequence alignment of UBL3, Ub, and other UBLs. The sequence homology of UBL3 to Ub and to other UBLs is relatively low (13.1–24.7%). Identical amino acids are highlighted in white (≤60%), light blue (61–80%) and mid-blue (81–100%). Clustal colored multiple sequence alignments were performed with Clustal Omega (https://www.ebi.ac.uk/Tools/msa/clustalo/) and displayed with Jalview version 2.11.0 (http://www.jalview.org/). ATG, autophagy-related protein; C (in the Ub-fold schema), carboxyl-terminal; C (in the UBL3 schema), cysteine; G, glycine; I, isoleucine; L, leucine; N, amino-terminal; NEDD8, neuronal precursor cell-expressed, developmentally down-regulated 8; SUMO1, small ubiquitin-like modifier 1; Ub, ubiquitin; UBL, ubiquitin-like protein; UBL3, ubiquitin-like 3; UFM1, ubiquitin-fold modifier 1; V, valine.

The primary structure of HsUBL3 consists of 117 amino acids. A Ub-fold domain encompassing residues 10 to 88 and a CAAX sequence (generally, where C is cysteine, A is an aliphatic amino acid and X is any amino acid) at the end of the carboxyl-terminal are two significant characteristics (Fig. 2a). The CAAX sequence of HsUBL3 consists of C, valine (V), isoleucine (I) and leucine (L). The secondary structure elements (α-helix and β-sheet) are arranged in the order ββαβββ (Fig. 2b) and form the “β-grasp” tertiary structure of the Ub-fold, in which the α-helix transverses a groove comprising an arched four-stranded β-sheet. This highly stable structure is shared in HsUb and other HsUBLs (Fig. 3a). The maturation of most UBLs (SUMO1, NEDD8, ATG8, ATG12 and UFM1) is promoted by digestion of the carboxyl-termini with carboxy-terminal hydrolases. This process exposes glycine residues which form isopeptide bonds with accessible lysine residues in the substrate protein. The three-enzyme cascade mediates these substrate protein modifications in the same manner as in Ub. Briefly, this cascade is initiated by E1, which forms a high energy thioester bond with Ub. The activated Ub forms a thioester bond with E2 to catalyze isopeptide bonding between Ub and the substrate protein. Although not universally required, E3 plays a role in identifying appropriate substrate proteins (Gavin *et al.*, 2014; Ichimura *et al.*, 2000; Kahyo *et al.*, 2001; Kirisako *et al.*, 2000; Mizushima *et al.*, 1998; Pang *et al.*, 2019; Tanaka *et al.*, 1998; Walden *et al.*, 2003). UBL3, Rad23 (Farmer *et al.*, 2010) and HUB (Dittmar *et al.*, 2002) are exceptions in this family as they contain no glycine residues in their carboxy-terminal region. In addition, the CAAX sequence of UBL3 is unique from other UBLs (Fig. 3a) (Dowil *et al.*, 2011).

UBLs are generally divided into the type-1 and type-2 subgroups. The general features of type-1 UBLs are small size, overall structural similarity with Ub, and their role as covalent modifiers of substrate proteins. Type-2 UBLs contain large domains that are structurally unrelated and have roles distinct from type-1 UBLs in protein-protein interactions without covalent modification (Hochstrasser, 2009; Tanaka *et al.*, 1998). SUMO1, NEDD8, ATG8, ATG12, and UFM1 are classified as type-1 UBLs, while Rad23 is classified as a type-2 UBL. HUB1 is an exception and differs from the two UBL subgroups; although it is small like type-1 UBLs and does not have the domain structure of large proteins, it does not conduct covalent modification, similar to type-2 UBLs (Yashiroda and Tanaka, 2004). UBL3 is small, has overall structural similarity with Ub and is involved in substrate protein modification via cysteine residues at the C-CAAX sequence, as described later. UBL3 is generally classified as a type-1 UBL.

**Fig. 4:**
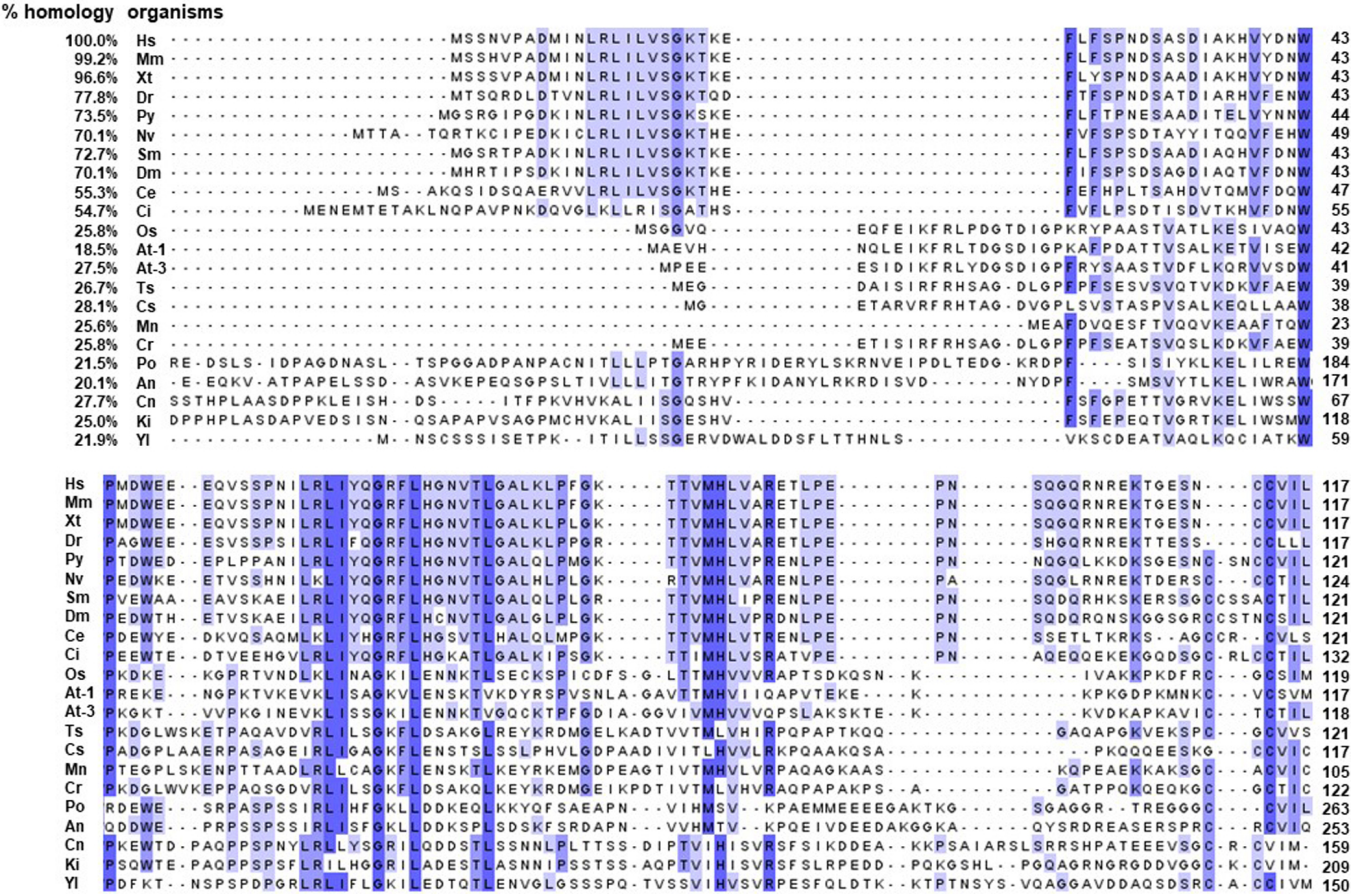
Multiple sequence alignments of UBL3 orthologs in various eukaryotes Primary structure homology of UBL3 orthologs in other eukaryotes to HsUBL3 varies widely (18.5–99.2%). Identical amino acids are highlighted in white (≤60%), light blue (61–80%) and mid-blue (81–100%). At-1 and At-3 are orthologs in *Arabidopsis thaliana* mentioned as AtMUB1 and AtMUB3, respectively by Downes *et al.* (Downes *et al.*, 2006). Clustal colored multiple sequence alignments were performed with Clustal Omega (https://www.ebi.ac.uk/Tools/msa/clustalo/) and displayed with Jalview version 2.11.0 (http://www.jalview.org/). The GenBank^TM^ accession numbers of the UBL3 orthologs are as follows: *Homo sapiens*, CAG38489; *Mus musculus*, BAE22201; *Xenopus tropicalis*, AAH44683; *Danio rerio*, NP_998021; *Patinopecten yessoensis*, XP_021348282; *Nematostella vectensis*, XP_001626774; *Stegodyphus mimosarum*, KFM60655; *Drosophila melanogaster*, NP_001162763; *Caenorhabditis elegans*, NP_001021222; *Ciona intestinalis*, XP_009860727; *Oryza sativa*, XP_015630437; *Arabidopsis thaliana*, NP_001319438 (At-1), NP_001329044 (At-3); *Tetrabaena socialis*, TSOC_00702; *Chlorella sorokiniana*, PRW20945; *Monoraphidium neglectum*, XP_013901616; *Chlamydomonas reinhardtii*, XP_001696591; *Pyricularia oryzae*, XP_003719682; *Aspergillus nidulans*, XP_662336; *Cryptococcus neoformans*, XP_567533; *Kockovaella imperatae*, XP_021868326; *Yarrowia lipolytica*, AOW02568. An, *Aspergillus nidulans*; At, *Arabidopsis thaliana*; C, carboxyl-terminal; Ce, *Caenorhabditis elegans*; Ci, *Ciona intestinalis*; Cn, *Cryptococcus neoformans*; Cr, *Chlamydomonas reinhardtii*; Cs, *Chlorella sorokiniana*; Dm, *Drosophila melanogaster*; Dr, *Danio rerio*; Hs, *Homo sapiens*; Ki, *Kockovaella imperatae*; Mm, *Mus musculus*; Mn, *Monoraphidium neglectum*; N, amino-terminal; Nv, *Nematostella vectensis*; Os, *Oryza sativa*; Po, *Pyricularia oryzae*; Py, *Patinopecten yessoensis*; Sm, *Stegodyphus mimosarum*; Ts, *Tetrabaena socialis*; UBL3, ubiquitin-like 3; Xt, *Xenopus tropicalis*; Yl, *Yarrowia lipolytica*.

Most of the secondary structure of UBL3 is in the Ub-fold domain. The amino acid sequence homologies of HsUb-fold superfamily members to HsUBL3 are low (13.1%–24.7%) (Fig. 3b). In contrast, the amino acid sequence homologies of UBL3 orthologs in various eukaryotes to HsUBL3 vary broadly from 18.5% to 99.2% (Fig. 4). For example, MmUBL3 has a high sequence homology of 99.2% with HsUBL3, with the only difference in the fourth amino acid residue (histidine in MmUBL3 and asparagine in HsUBL3). In contrast, the lowest sequence homology of *Arabidopsis thaliana* (At) UBL3 with HsUBL3 is 18.5%.

**Fig. 5:**
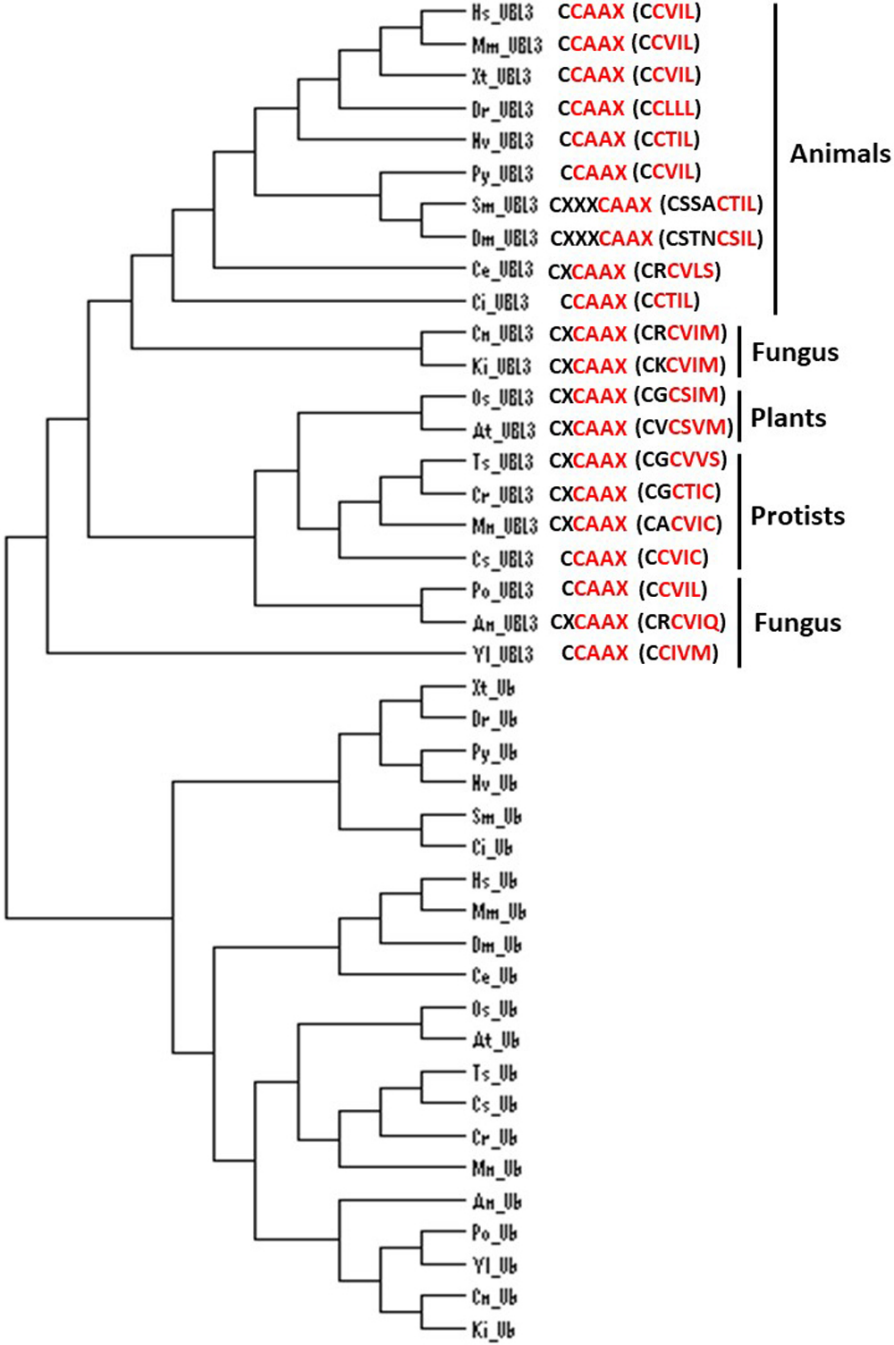
Phylogenic analysis of UBL3 orthologs in various eukaryotes, focusing on the CAAX sequence at the carboxyl-terminal and nearby cysteine residues The UBL3-ortholog group was separated from the Ub-out-group. Two adjacent cysteine residues are observed in animals, except for *Caenorhabditis elegans*, and the CAAX sequence (colored red) is evolutionarily conserved in organisms at various stages of evolution. Phylogenic analysis was performed with Clustal Omega (https://www.ebi.ac.uk/Tools/msa/clustalo/) and was drawn using Dendroscope 3 version 3.6.2 (http://ab.inf.uni-tuebingen.de/software/dendroscope/). The GenBank^TM^ accession numbers of the UBL3 orthologs mutual with Fig. 4 are used, while that of the Ub orthologs and the range of amino acid (AA) sequences used in the analysis are as follows: *Homo sapiens*, 1C3T_A (full length); *Mus musculus*, NP_001335156 (1-76AA); *Xenopus tropicalis*, NP_001005136 (1-74AA); *Danio rerio*, NP_957031 (1-74AA); *Patinopecten yessoensis*, XP_021343069 (1-72AA); *Nematostella vectensis*, XP_001625352 (1-74AA); *Stegodyphus mimosarum*, KFM61924 (1-72AA); *Drosophila melanogaster*, AAA28999 (full length); *Caenorhabditis elegans*, NP_499695 (1-76AA); *Ciona intestinalis*, XP_009859868 (1-74AA); *Oryza sativa*, XP_015629795 (1-76AA); *Arabidopsis thaliana*, NP_565836 (1-76AA); *Tetrabaena socialis*, PNH01953 (1-76AA); *Chlorella sorokiniana*, PRW56465 (1-76AA); *Monoraphidium neglectum*, XP_013899085 (1-76AA); *Chlamydomonas reinhardtii*, XP_001700313 (1-76AA); *Pyricularia oryzae*, XP_003711895 (1-76AA); *Aspergillus nidulans*, FAA00317 (1-76AA); *Cryptococcus neoformans*, XP_018996320 (1-76AA); *Kockovaella imperatae*, XP_021870639 (1-76AA); *Yarrowia lipolytica*, XP_505175 (1-76AA). An, *Aspergillus nidulans*; At, *Arabidopsis thaliana*; C, carboxyl-terminal; Ce, *Caenorhabditis elegans*; Ci, *Ciona intestinalis*; Cn, *Cryptococcus neoformans*; Cr, *Chlamydomonas reinhardtii*; Cs, *Chlorella sorokiniana*; Dm, *Drosophila melanogaster*; Dr, *Danio rerio*; Hs, *Homo sapiens*; Ki, *Kockovaella imperatae*; Mm, *Mus musculus*; Mn, *Monoraphidium neglectum*; N, amino-terminal; Nv, *Nematostella vectensis*; Os, *Oryza sativa*; Po, *Pyricularia oryzae*; Py, *Patinopecten yessoensis*; Sm, *Stegodyphus mimosarum*; Ts, *Tetrabaena socialis*; Ub, ubiquitin; UBL3, ubiquitin-like 3; Xt, *Xenopus tropicalis*; Yl, *Yarrowia lipolytica*.

**Fig. 6:**
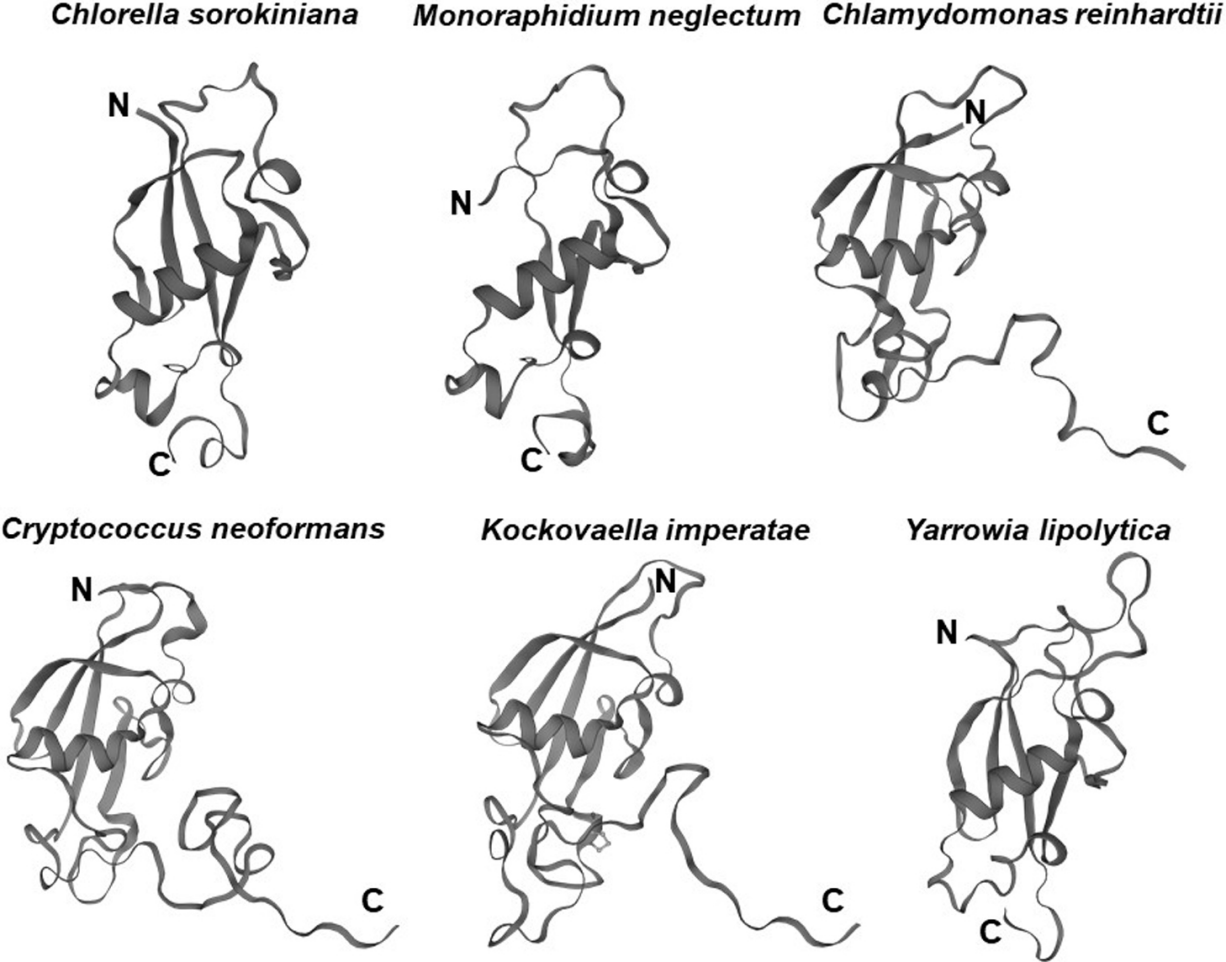
Tertiary structures of UBLs harboring the CAAX sequence in unicellular organisms UBLs with a Ub-fold and the unstructured carboxyl-terminal in unicellular algae (*Chlorella sorokiniana*, *Monoraphidium neglectum*, *Chlamydomonas reinhardtii*) and fungi (*Cryptococcus neoformans*, *Kockovaella imperatae*, *Yarrowia lipolytica*) are shown. Three-dimensional diagrams were drawn using SWISS-MODEL (https://swissmodel.expasy.org/) and the amino acid sequences obtained from the following GenBank^TM^ accession numbers: *Chlorella sorokiniana*, PRW20945; *Monoraphidium neglectum*, XP_013901616; *Chlamydomonas reinhardtii*, XP_001696591; *Cryptococcus neoformans*, XP_567533; *Kockovaella imperatae*, XP_021868326; *Yarrowia lipolytica*, AOW02568. C, carboxyl-terminal; Cn, *Cryptococcus neoformans*; Cr, *Chlamydomonas reinhardtii*; Cs, *Chlorella sorokiniana*; Ki, *Kockovaella imperatae*; Mn, *Monoraphidium neglectum*; N, amino-terminal; Ub, ubiquitin; UBL, ubiquitin-like protein; Yl, *Yarrowia lipolytica*.

The *UBL3* gene has not been identified in eukaryote genomes such as *Schizosaccharomyces pombe* and *Saccharomyces cerevisiae* (Chadwick *et al.*, 1999), and thus UBL3 has been believed to be exclusive to multicellular organisms (Dowil *et al.*, 2011; Downes *et al.*, 2006). Interestingly, a Ub-like protein in *Tetrabaena socialis* (Ts) can be found as “membrane-anchored ubiquitin-fold protein (MUB) 4” by a BLAST search (https://blast.ncbi.nlm.nih.gov/Blast.cgi) using AtUBL3 as a query sequence. *Tetrabaena socialis* is a four-celled green alga member of the most basal group of multicellular organisms (Arakaki *et al.*, 2013). TsMUB4 has the CAAX sequence at its carboxyl-terminal and is thus a potential UBL3 ortholog. Also, potential UBL3 orthologs harboring the Ub-like domain and the CAAX sequence in more primitive unicellular green algae, such as Chlorella sorokiniana (Cs), Monoraphidium neglectum (Mn) and Chlamydomonas reinhardtii (Cr), can be listed by BLAST searches using the TsMUB4 sequence as the query. In the same manner, potential UBL3 orthologs in unicellular fungi, such as Cryptococcus neoformans (Cn), Kockovaella imperatae (Ki), and Yarrowia lipolytica (Yl), can be listed by using the sequence of Aspergillus nidulans (An) UBL3 as a query. The amino acid sequence homologies of these Ub-like proteins to HsUBL3 vary from 21.9% to 27.7% (Fig. 4). The UBL3 orthologs, including these potential candidates in unicellular organisms, arranged in a phylogenic tree, were separated from the Ub out-group (Fig. 5). Furthermore, tertiary structure prediction using SWISS-MODEL (https://swissmodel.expasy.org/) shows the Ub-fold in all these UBLs from unicellular organisms (Fig. 6). Although the roles of these UBLs with a CAAX sequence in unicellular organisms have not been elucidated, they are likely potential UBL3 orthologs.

UBL3 orthologs, including the possible candidates, noted above, retain the CAAX sequence, showing that this sequence is evolutionarily conserved (Fig. 4). In HsUBL3, the 113th residue (C113) directly connects to the CAAX sequence (C-CAAX), and thus there are two cysteine residues next to each other (C113C114VIL). As discussed in detail later, these two consecutive cysteine residues are involved in protein modification (C113) and prenylation (C114) in Hs/MmUBL3 (Ageta *et al.*, 2018). Although the cysteine residue of the CAAX sequence of AtUBL3 is known to engage in membrane anchoring via prenylation (Downes *et al.*, 2006), whether AtUBL3 undergoes protein modification through the cysteine residue near the CAAX sequence remains unknown. Focusing upstream of the CAAX sequence in UBL3 orthologs arranged in the phylogenic tree, UBL3 orthologs in all the indicated organisms have a cysteine residue upstream of the CAAX sequence (Fig. 5). Furthermore, the arrangement of the cysteine residues is different for each biological kingdom. For example, two cysteine residues are located consecutively (C-CAAX) in *Homo sapiens*, *Mus musculus*, *Xenopus tropicalis* (Xt), *Danio rerio* (Dr), Nematostella vectensis (Nv), *Patinopecten yessoensis* (Py), and *Ciona intestinalis* (Ci). The arthropods (*Stegodyphus mimosarum* [Sm] and *Drosophila melanogaster*) contain the CXXXCAAX sequence, in which three amino acid residues (of any type) are inserted between the CAAX sequence and the nearest cysteine residue located upstream. Most plants, including plant protists (*Oryza sativa* [Os], *Arabidopsis thaliana*, *Tetrabaena socialis*, *Chlamydomonas reinhardtii*, *Monoraphidium neglectum*), have a CXCAAX sequence, whereas fungi have CCAAX or CXCAAX. Therefore, the arrangement of cysteine residues near the CAAX sequence may reflect the biological classification in the phylogenetic tree. Further studies will likely elucidate whether protein modification and membrane anchoring via these cysteine residues are also evolutionally conserved.

**Fig. 7:**
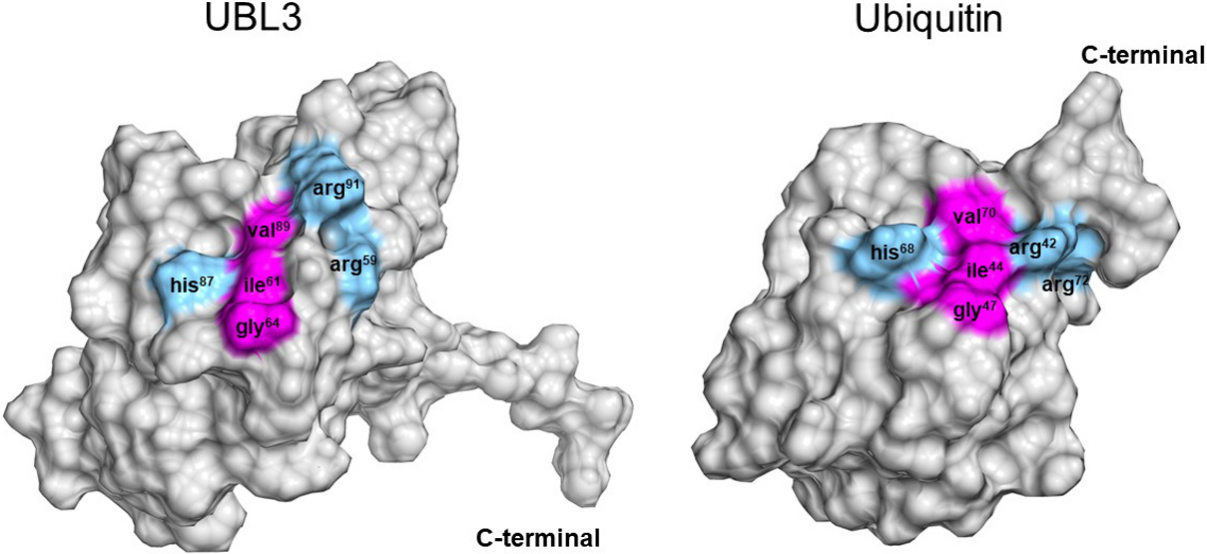
Isoleucine-centered hydrophobic patch conserved in UBL3 and Ub The isoleucine44-centered hydrophobic patch of HsUb corresponds to the isoleucine61-centered hydrophobic patch in HsUBL3, and the associated residues are also well conserved. These hydrophobic patches are the noncovalent binding sites for UBDs. Hydrophobic and basic residues are indicated in magenta and light blue, respectively. Three-dimensional diagrams of the Ub/UBLs were drawn using QueMol 2.0 (http://www.cuemol.org/ja/) and the following Protein Data Bank IDs (http://www.rcsb.org/): human UBL3, 2GOW; human Ub, 1C3T. arg, arginine; gly, glycine; his, histidine; Hs, *Homo sapiens*; ile, isoleucine; Ub, ubiquitin; UBD, ubiquitin binding domain; UBL3, ubiquitin-like 3; val, valine.

Another characteristic tertiary structure of UBL3 is an isoleucine-centered hydrophobic patch located within the Ub-fold (Fig. 7). Norberto *et al.* conducted an NMR analysis of the HsUBL3 structure and found an isoleucine-centered hydrophobic patch that acts as a noncovalent interaction site for α-helical Ub-binding domains (UBDs) (Hurley *et al.*, 2006). This hydrophobic patch is well conserved in Ub and NEDD8, and is isoleucine61- and isoleucine44-centered in human UBL3 and Ub, respectively. The noncovalent interaction between this hydrophobic patch and UBDs plays an essential role in substrate protein modification through the three-enzymatic cascade (E1, E2 and E3) in Ub and UBLs. Therefore, some molecules harboring the α-helical UBD may interact non-covalently with HsUBL3 to regulate its physiological role.

## The role of UBL3 in cellular physiology

### 1. UBL3 as a regulator of the Ub-system in plants

UBL3 orthologues in *Arabidopsis thaliana* were characterized in 2006 as membrane-anchored proteins, referred to as MUBs (Downes *et al.*, 2006). In addition, that study showed that the CAAX sequence of UBL3 functions as a membrane-anchoring signal through prenylation of its cysteine residue.

The physiological role of UBL3 in *Arabidopsis thaliana* was partly elucidated between 2011 and 2016 by a series of studies. An isoleucine-centered hydrophobic patch on AtUBL3 was shown to non-covalently interact with UBDs in a specific subgroup of AtE2, resulting in the E2s localizing to the plasma membrane and demonstrating that UBL3 regulates the Ub-cascade by inhibiting subsequent E2-Ub formation (Dowil *et al.*, 2011; Lu *et al.*, 2016).

### 2. Rediscovery of UBL3 as a novel protein-sorting factor to sEVs via PTM in mammals

Although aspects of UBL3 function had been revealed in plants, the most significant roles of UBL3 in cellular physiology in both plants and animals remained largely obscure. In 2018, a revolutionary concept was set forth by Ageta and colleagues: it proposed that UBL3 acts as a novel PTM factor that promotes protein sorting to sEVs in animals (Ageta *et al.*, 2018). They elucidated the physiological role of UBL3 by focusing on its CAAX sequence. In the MDA-MB-231 human breast cancer cell line, PTM of substrate proteins by UBL3 was shown to depend on cysteine residues in the C-CAAX sequence (C113/C114) of UBL3. Furthermore, UBL3 was found to be packaged within sEVs purified from MDA-MB-231 cells, and vesicles within MVBs are released into the extracellular space as sEVs through the fusion of MVBs to the plasma membrane (Simons and Raposo, 2009); thus, they concluded that UBL3 contributes to the extracellular secretion of sEV-proteins. They subsequently identified 1241 proteins interacting with the two cysteine residues (C113/114) of UBL3. These UBL3-interacting proteins included at least 22 molecules involved in oncogenesis, tumor proliferation/invasion/metastasis, neurodegenerative/neuronal diseases, immune response, and mTOR/Notch/BMP signaling. Sorting of oncogenic-Ras protein, one of the UBL3 interacting proteins, to sEVs was promoted by UBL3, and this Ras-containing sEVs activated Ras-signaling in recipient cells. Thus, UBL3 was demonstrated to participate in cell-to-cell communication via protein sorting to sEVs.

Prior to this study, Ub-proteasome and ATG8/ATG12-autophagy systems were believed to be the primary protein sorting systems involving PTM by Ub and UBL. The paradigm shift regarding the function of UBL3 elevated it to the same level of importance as Ub-proteasome and ATG8/ATG12-autophagy systems (Fig. 1). Meanwhile, UBL3 holds promise as a therapeutic target for sEV-related diseases and may find application as DDS cargo.

## How does UBL3 promote protein sorting to sEVs?

UBL3 was shown to promote protein sorting to sEVs by its C-CAAX sequence (CCVIL). This C-CAAX sequence plays two essential roles: membrane localization of UBL3 (Dowil *et al.*, 2011; Downes *et al.*, 2006; Lu *et al.*, 2016) and PTM of substrate proteins (Ageta *et al.*, 2018). This section discusses a possible mechanism by which UBL3 sorts proteins into sEVs, focusing on these two essential roles of the C-CAAX sequence.

### 1. The role of the CAAX sequence on the membrane localization of UBL3

A detailed understanding of the membrane localization of UBL3 due to its C-CAAX sequence is lacking. Thus, we discuss an analogous mechanism for Ras proteins, a family of well-studied CAAX proteins, in conjunction with previous UBL3 studies.

Ras family members are representative CAAX proteins. They localize to the plasma membrane after CAAX prenylation and can be activated to generate downstream signals (Wang and Casey, 2016; Wright and Philips, 2006). Ras family members typically mature upon modification of the CAAX sequence via three enzymatic steps: 1) prenylation of the cysteine residue by protein geranylgeranyltransferase type 1 (PGGTI) or farnesyl transferase (FT), 2) proteolytic processing (removal of AAX tripeptides) by Rce1, and 3) carboxylmethylation of the cysteine residue by isoprenylcysteine carboxyltransferase (IMCT) (Wang and Casey, 2016). Rce1 is a post-prenylation protease active towards most human CAAX proteins (Hampton *et al.*, 2018; Manolaridis *et al.*, 2013; Wang and Casey, 2016). Based on limited information from previous UBL3 studies, the CAAX sequence (CTIL) in one of the UBL3 orthologs in *Arabidopsis thaliana* (shown as At-3 in Fig. 4) is likely modified by geranylgeranylation rather than farnesylation (Downes *et al.*, 2006). This is plausible because the cysteine residue in the CAAX sequence, where X is an L residue, tends to be targeted by PGGTI (Andrews *et al.*, 2010; Randall *et al.*, 1993). Therefore, the cysteine residue in the CAAX sequence of HsUBL3, “CVIL”, may be preferentially modified with geranylgeranyl moieties. In addition, Downes found that AtUBL3 with the wild type CAAX sequence expressed in *Arabidopsis thaliana* migrated faster during SDS-PAGE than a mutated CAAX sequence (C114S: SAAX). This finding is consistent with adding a prenyl-group and subsequent proteolytic processing (Downes *et al.*, 2006).

### 2. The role of the CAAX sequence on protein PTM

The PTM of substrate proteins via the cysteine residues at the carboxyl-terminal has been extensively studied. Lu *et al.* mentioned that UBL3 orthologs in *Arabidopsis thaliana* are significantly different from Ub, SUMO and NEDD8, in that the UBL3 orthologs are not protein modifiers (Lu *et al.*, 2016). However, Ageta *et al.* demonstrated that the cysteine residues at the carboxyl-terminal of Hs/MmUBL3 are responsible for the PTM of substrate proteins (Ageta *et al.*, 2018) and showed that C113 mutation drastically decreased the UBL3 modification of substrate proteins compared to C114 mutation. Besides, C114 mutation decreased membrane localization more than C113 mutation (Ageta *et al.*, 2018). Furthermore, Downes demonstrated that prenylated C114 is responsible for membrane localization in AtUBL3 (Downes *et al.*, 2006). In Hs/MmUBL3, C113 is thus considered to be intensely involved in PTM and C114 to be preferentially geranylgeranylated. In addition, deletion of the leucine residue at the end of the CAAX sequence (CVIL) prevents UBL3 modification (Ageta *et al.*, 2018), and thus substrate protein modification and prenylation must occur before removal of the tripeptide (VIL). Details of the bonding between UBL3 and the substrate protein remain unknown. However, the bonding is believed to be regulated by a redox reaction because bonding is inhibited under reducing conditions in the presence of 2-mercaptoethanol (Ageta *et al.*, 2018). Covalent bonding by cysteine residues to form a disulfide or thioester bond is inhibited under reducing conditions. A redox reaction regulates disulfide bond formation, while, thioester bond formation requires adenosine triphosphate because it is a “high-energy” bond. The bond formation mechanism will be determined in the future by mass spectrometry.

### 3. UBL3 protein sorting model: an anticipated outlook

**Fig. 8:**
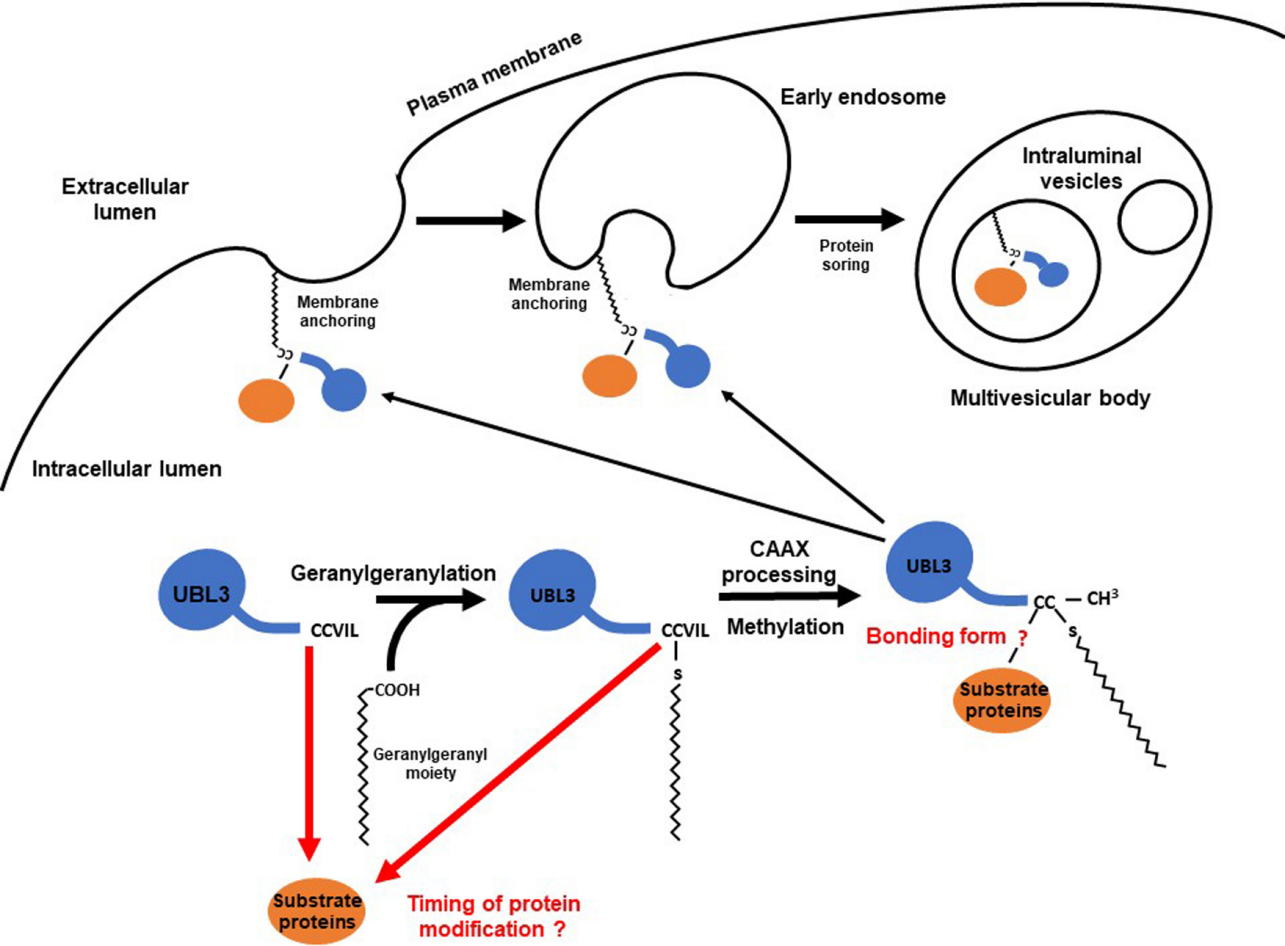
The proposed UBL3 protein sorting model Post-translational modification of substrate proteins may occur before CAAX processing, although the precise timing of this process is unknown. UBL3 with a geranylgeranylated cysteine residue is anchored to the plasma membrane or MVBs. Substrate proteins are then sorted into intraluminal vesicles together with UBL3. The bonding structure between UBL3 and the substrate protein is unknown. MVBs, multivesicular bodies; UBL3, ubiquitin-like 3.

Assuming that the C-CAAX sequence of HsUBL3 and MmUBL3 (CCVIL) are modified as described above, UBL3 may promote protein sorting to sEVs as follows (Fig. 8):

(1) C113 of UBL3 in the cytosol modifies substrate proteins.

(2) C114 of UBL3 is geranylgeranylated by PGGTI. Because the timing of target protein modification is unknown, it is also possible that UBL3 is first geranylgeranylated, followed by PTM of the substrate protein by UBL3.

(3) The tripeptide (VIL) of the CAAX sequence is proteolytically removed by Rce1.

(4) The geranylgeranylated C113 is carboxymethylated by IMCT.

(5) The protein modified with mature UBL3 is anchored to the plasma membrane or early endosomes by the geranylgeranyl-moiety attached to the C114.

(6) The membrane-anchored substrate protein is transported to ILVs in the MBVs, in association with the inward curvature of the early endosome.

When MVBs fuse to the plasma membrane, ILVs are released as sEVs to the extracellular space.

### 4. Known mechanisms of protein sorting to sEVs other than UBL3

Known mechanisms of protein sorting to sEVs other than UBL3 can be primarily divided into the endosomal sorting complex required for transport (ESCRT)-dependent and ESCRT-independent pathways (Katzmann, 2006; van Niel *et al.*, 2011). The ESCRT pathway is an evolutionarily conserved ancient system in animals, plants, yeast, archaebacteria and viruses (Gao *et al.*, 2017; Wollert *et al.*, 2009).

In the ESCRT-dependent pathway, protein sorting is promoted by four ESCRT complexes (ESCRT-0, I, II and III), which comprise more than 20 proteins. The ESCRT-dependent pathway is divided into Ub-dependent and Ub-independent pathways (Frankel and Audhya, 2018; Hurley, 2008; Katzmann *et al.*, 2001). In the Ub-dependent pathway, plasma membrane proteins are sorted parallel with MVB maturation by the following mechanism. First, plasma membrane proteins are ubiquitylated, and isoleucine44-centered in the hydrophobic patch of Ub is recognized by the UBDs of ESCRT-0, I, II. The endosomal membrane is deformed, and ESCRT-III breaches the endosomal membrane to form ILVs, resulting in ubiquitylated protein sorting to ILVs (Frankel and Audhya, 2018; Gruenberg and Stenmark, 2004; Hurley, 2008; Piper and Katzmann, 2007). In contrast, in the Ub-independent pathway, plasma membrane proteins are directly recognized by ESCRT-associated proteins such as ALIX and Hrs without ubiquitination. For example, G protein-coupled protease-activated receptor-1 and purinergic receptor P2Y_1_ are recognized by ALIX. Also, interleukin-2 receptor β is recognized by Hrs. Downstream of the cargo sorting processes via ALIX and Hrs follow the same process as the ESCRT pathway (Frankel and Audhya, 2018). SUMOylation occurs via a Ub-independent pathway. Extracellular accumulation of α-synuclein in the brain is a histopathological hallmark of Parkinson’s disease. SUMOylation is known to promote the interneuronal propagation of α-synuclein by sorting it into sEVs (Kunadt *et al.*, 2015). The heterogeneous nuclear ribonucleoprotein A2B1 (hnRNP2B1) is also SUMOylated, then sorted into sEVs. The miRNAs are sorted into sEVs by hnRNP2B1 binding to miRNAs, which SUMOylation controls, and thus miRNA sorting is indirectly regulated by the SUMOylation of hnRNP2B1 (Villarroya-Beltri *et al.*, 2013). In these pathways, SUMOylated proteins are believed to be localized to the ILV formation site due to the hydrophobic cleft and nearby N-terminal loop of SUMO binding to phospholipids in the plasma membrane. Subsequently, the targeted protein is sorted to MVBs by the ESCRT pathway (Kunadt *et al.*, 2015).

Several studies have shown that inhibition of the ESCRT pathway decreases protein sorting to MVBs (Colombo *et al.*, 2013; Tamai *et al.*, 2010). However, some physiologically critical proteins are sorted into MVBs independent of ubiquitination or the ESCRT pathway (Buschow *et al.*, 2009; Trajkovic *et al.*, 2008). For example, tetraspanin CD63 is an ESCRT-independent protein sorting pathway which promotes premelanosome protein sorting into MVBs in melanocytes (van Niel *et al.*, 2011). In this pathway, MVB formation is believed to be promoted by ceramides which induce inward curvature of the MVB membrane (Trajkovic *et al.*, 2008).

Although the detailed mechanism of protein sorting to the ILVs of MVBs by UBL3 remains unknown, substrate proteins modified with UBL3 may be sorted into the ILVs of MVBs by cross-talk with the UBDs of ESCRT proteins, given that 1) the isoleucine-centered hydrophobic patch (the interaction site for UBDs) is conserved in UBL3 (de la Cruz *et al.*, 2007; Dowil *et al.*, 2011), and 2) both STAM1 and STAM2, which are ESCRT-0 components that harbor UBDs (Frankel and Audhya, 2018; Hong *et al.*, 2009; Lange *et al.*, 2012), are found in UBL3-interacting proteins (Ageta *et al.*, 2018). Alternatively, UBL3 may be an ESCRT-independent pathway for protein sorting. Further studies are required to elucidate the detailed mechanism of protein sorting by UBL3.

## Future perspectives for UBL3 investigations in human disease and therapy

### 1. UBL3 and sEV-related diseases

In this section, we discuss the potential of UBL3 as a therapeutic target for sEV-related diseases and as a cargo loading strategy for engineering sEVs as DDS.

sEVs carry complex cargo consisting of proteins, lipids, and nucleic acids and serve as functional mediators for cancer progression and neurodegenerative disease by delivering cargo to recipient cells (Simons and Raposo, 2009). For example, various cargo proteins contained in sEVs promote each step of cancer progression (Fujita *et al.*, 2016; Steinbichler *et al.*, 2017), and sEV-targeting therapies have been reported to inhibit cancer progression (Nishida-Aoki *et al.*, 2017). sEVs have also been found to promote the propagation of Tau protein and β-amyloid (Aβ) peptides in the brain, leading to Alzheimer’s disease (AD) (Asai *et al.*, 2015; Rajendran *et al.*, 2006). UBL3-interacting proteins (Ageta *et al.*, 2018) are associated with sEV-related diseases. Thus, regulating UBL3 which promotes cargo protein loading to sEVs is a promising therapeutic target for delaying cancer progression and treating neurodegenerative diseases.

The development phase of UBL3-regulating agents will require understanding the mechanism of bonding between UBL3 and substrate proteins and identification of the enzymes that promote or reverse UBL3 modification. The total protein content of sEVs in *UBL3*-knock out mice established by Ageta and colleagues was 60% lower than wild-type mice (Ageta *et al.*, 2018). If no critical phenotype for survival is identified by further analysis of *UBL3*-KO mice, the potential for critical side effects induced by inhibiting UBL3 may be low.

Research on the application of sEVs as DDS by engineering the cargo loading has made recent progress (Antimisiaris *et al.*, 2018; Johnsen *et al.*, 2014). Ageta and colleagues proposed that UBL3 could be a valuable tag for therapeutic agents loaded into sEVs (Ageta *et al.*, 2018; Ageta and Tsuchida, 2019).

Below, we describe UBL3-interacting proteins and highlight the involvement of each in cancer progression and neurodegenerative disease. Furthermore, we indicate the relationship between UBL3 and these sEV-related diseases. In addition, we describe the possible use of UBL3 in DDS development.

### 2. Relationship between UBL3 and cancer progression

**Fig. 9:**
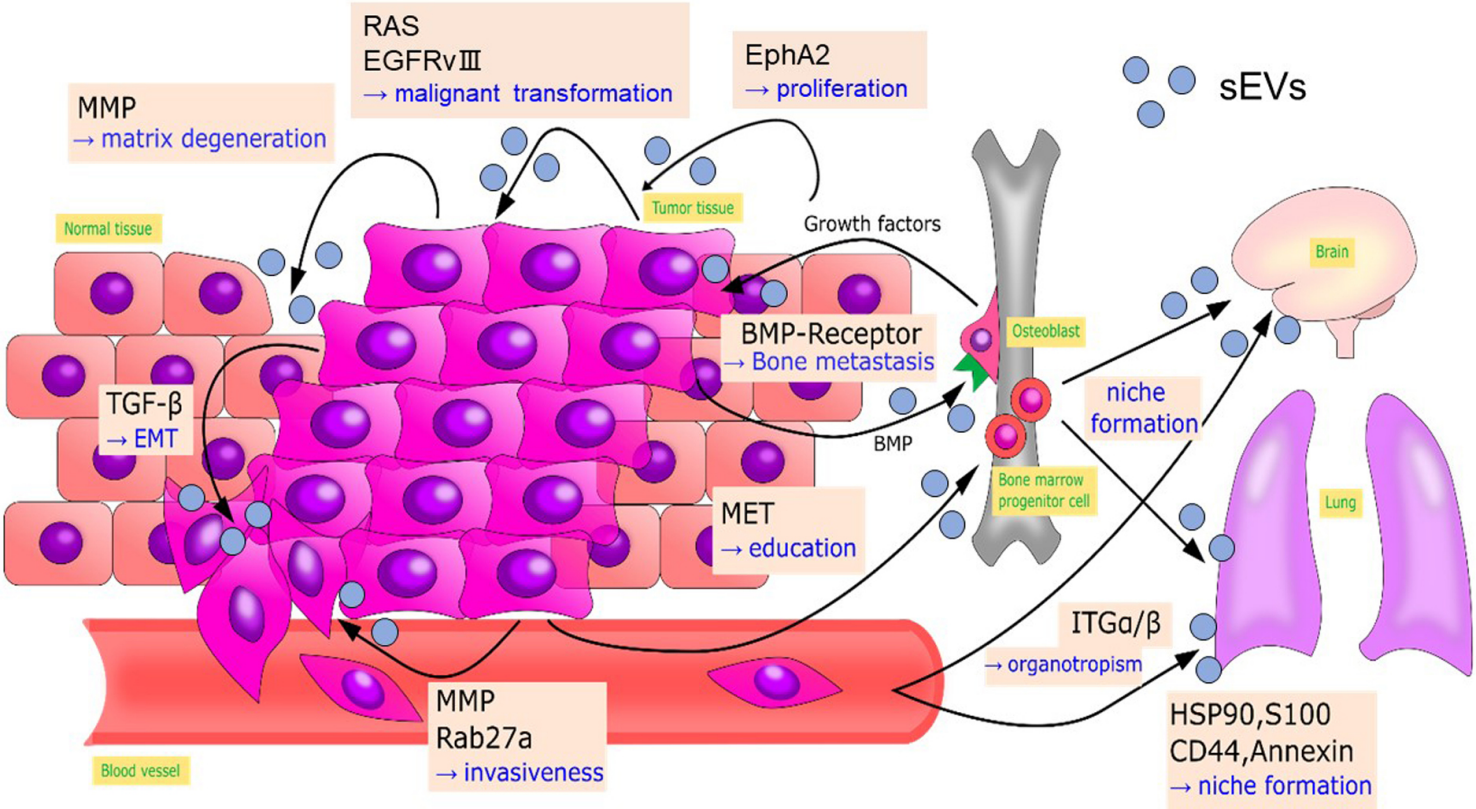
Cancer progression-related sEV-proteins that interact with UBL3 Multistep processes of cancer progression, showing sEV-proteins that interact with UBL3. Arrows indicate sEV delivery. BMP, bone morphogenic proteins; EGFRvIII, epidermal growth factor receptor variant III; EMT, epithelial to mesenchymal transition; HSP90, heat shock protein 90; ITG, integrin; MMT, matrix metalloprotease; sEV, small extracellular vesicle; TGF-β, tumor growth factor-β; UBL3, ubiquitin-like 3.

Cancer progression consists of multiple processes, including malignant transformation, surrounding tissue invasion, blood and lymphatic vessel invasion, attachment to host organs, and the formation of metastatic lesions. Each cargo protein in cancer cell or cancer stromal cell-derived sEVs has particular roles in the steps in cancer progression (Fujita *et al.*, 2016; Steinbichler *et al.*, 2017). Here, we introduce UBL3-interacting proteins that are sorted to sEVs derived from cancer cells or cancer stromal cells and reported to be involved in cancer progression (Ageta *et al.*, 2018) (Fig. 9).

Gene expression of cancer cells within an identical cancer tumor is heterogeneous. Cancer cells communicate by transferring components for malignant transformation via sEVs. For example, functional epidermal growth factor receptor variant III in glioblastoma is transferred among cancer cells by sEVs, leading to malignant transformation (Al-Nedawi *et al.*, 2008). Oncogenic Ras protein is transferred to recipient cells (Ageta *et al.*, 2018). Also, EphA2 sorted in sEVs derived from senescent cancer stromal cells promotes cell proliferation by activating EphA2/ephrin-A1 reverse signaling in recipient cancer cells (Takasugi *et al.*, 2017).

Epithelial to mesenchymal transition (EMT) is an initial step in metastasis. This step initiates tumor cell migration into blood vessels or lymphatic vessels by acquiring mesenchymal characteristics, such as loss of polarity, loss of cell-cell junctions, enhancement of migratory capability and invasion capabilities (Steinbichler *et al.*, 2017). Tumor growth factor-β in sEVs derived from bladder cancer and lung cancer cells induce EMT by decreasing the expression of E-cadherin and β-catenin that stabilize proteins at cell-cell junctions (Franzen *et al.*, 2015; Rahman *et al.*, 2016).

Tumor-derived sEVs can also remodel the microenvironment around the tumor to aid cancer cell invasion. In a hypoxic environment, A431 carcinoma cells secrete matrix metalloproteases (MMPs) via sEVs that degenerate extracellular matrix to enhance tumor cell invasion (Park *et al.*, 2010). MMPs also stimulate tumor invasion and metastasis in sarcoma and nasopharyngeal cancers (Min *et al.*, 2016; You *et al.*, 2015). RAB27a and RAB27b, which are regulators for sEV secretion (Ostrowski *et al.*, 2010), enhance the invasiveness and metastasis of breast cancer by promoting the secretion of MMPs, heat shock protein 90 (HSP90), and other mediators of cancer progression (Hendrix *et al.*, 2010; Wang *et al.*, 2008). Bone morphogenetic proteins secreted by prostate cancer cells via sEVs stimulate osteoblast proliferation, promoting the deposition of new bone matrix. Stimulated osteoblasts are believed to produce growth factors that in turn stimulate the proliferation of prostate cancer cells and subsequent bone metastasis (Logothetis and Lin, 2005).

Neoangiogenesis facilitates the supply of nutrients and oxygen to tumor cells, allowing them to grow and invade past the diffusion limit of oxygen. This process plays an essential role in the entry point of metastasis. During angiogenesis, MMPs are essential for the basement membrane’s breakdown and endothelial cell proliferation (Chang and Werb, 2001).

In 1989, Stephen Paget proposed the “seed and soil” hypothesis, in which metastatic cancer cells act as “seeds” and organs (the destination for metastasis) serve as the “soil” (Paget, 1989). Since then, target organs for cancer metastasis have been shown to be optimized (“niche formation”) prior to actual metastasis (Bidard *et al.*, 2008), and sEVs play essential roles in “niche formation” (Hood *et al.*, 2011; Peinado *et al.*, 2012). In melanoma, tumor-derived sEVs transfer MET oncoprotein to the bone marrow to guide progenitor cells toward a pro-vasculogenic and pro-metastatic phenotype, thus promoting pre-metastatic niche formation. sEVs isolated from highly metastatic melanoma cell lines have enhanced expression of HSP90, S100, CD44 and annexin, which are involved in metastatic niche formation (Peinado *et al.*, 2012). Tumor-derived sEVs also direct metastatic organotropism in niche formation. Hoshino *et al.* demonstrated that sEVs derived from breast cancer cells with specific metastatic organotropism express specific patterns of integrins which determine their organotropism: integrins α6β4 and α6β1 are responsible for lung metastasis. In contrast, integrin αvβ5 is related to liver metastasis (Hoshino *et al.*, 2015).

All the above cancer progression-related proteins are UBL3-interacting proteins (Ageta *et al.*, 2018), suggesting the importance of future studies of anti-cancer therapies targeting UBL3.

**Fig. 10:**
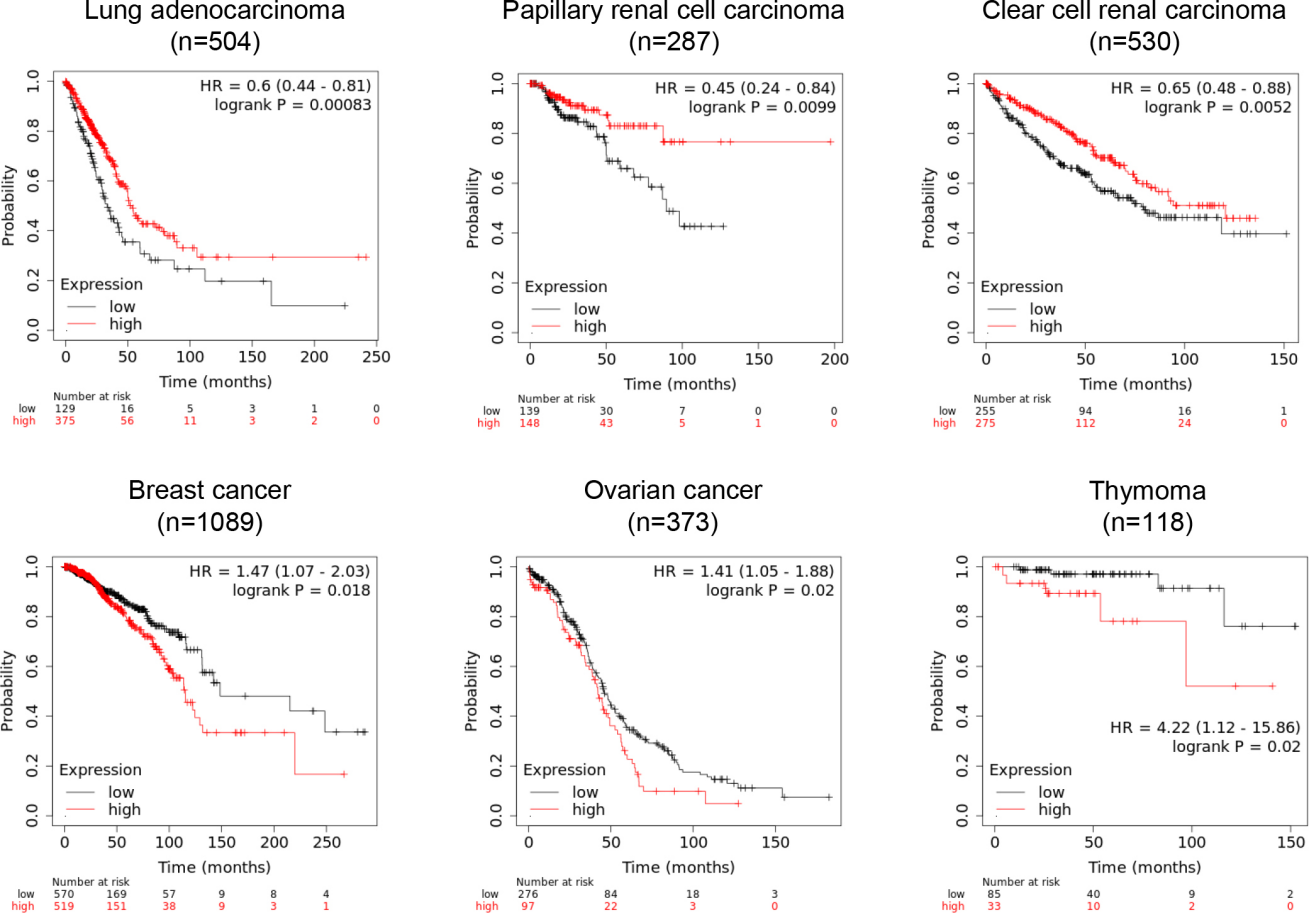
The prognostic significance of UBL3-mRNA expression on various malignant tumor types The relationship between UBL3-mRNA expression level and patient prognosis depends on the tumor type. High and low UBL3-mRNA expression groups are shown as red and black lines, respectively. Overall survival analysis for UBL3-mRNA expression was performed using datasets in The Cancer Genome Atlas research network (http://cancergenome.nih.gov). Kaplan-Meier plots, hazard ratio, 95% confidence intervals and log-rank *P* values were generated on Kaplan-Meier Plotter (https://kmplot.com/analysis/). Best cutoff values for discriminating good or poor prognosis groups were auto-selected. Tumor types with significant *P* values <0.05 are presented. Hazard ratio, HR; UBL3, ubiquitin-like 3.

An mRNA expression database shows the prognostic significance of UBL3-mRNA expression on various malignant tumor types. Intriguingly, the relationship between the UBL3-mRNA expression level and patient prognosis is different among tumor types. High UBL3-mRNA expression is associated with more prolonged overall survival for patients with lung adenocarcinoma, papillary and clear cell renal carcinoma (Fig. 10, upper panels). As UBL3 was demonstrated to act as a tumor suppressor in lung adenocarcinoma cell lines (Zhao *et al.*, 2020), promoting UBL3 activity in these cancer types may lead to new cancer treatment. In contrast, low UBL3-mRNA expression is associated with more prolonged overall survival for breast cancer, ovarian cancer, and thymoma (Fig. 10, lower panels), suggesting that these tumor types might be treated by inhibiting UBL3 activity.

It is difficult to explain the mechanism underlying these two contradictory possible therapeutic approaches targeting UBL3. One possible explanation is that inhibiting UBL3 is a therapeutic strategy for cancer types in which UBL3 promote invasion, metastasis, and malignant transformation of adjacent tumor cells by propagating cancer progression-related molecules via sEVs. In this case, inhibiting substrate protein modification through the CAAX sequence, or inhibiting a potential noncovalent bonding site for UBDs (the isoleucine61-centered hydrophobic patch), could provide methodologies for inhibiting UBL3 activity. Indeed, several therapeutic approaches for cancers targeting CAAX sequence processing on RAS proteins, such as the inhibition of PGGTI, FT, Rce1 and IMCT, have demonstrated anti-tumor effects (Wang and Casey, 2016). On the other hand, cancer types in which progression depends on the intracellular accumulation of malignant transformation factors or growth factors might be treated by discharging these factors into sEVs by promoting UBL3 activity. A methodology for promoting UBL3 activity can be formulated by assuming the existence of deconjugation enzymes for UBL3 and inhibiting their activities. Deubiquitinase and sentrin/SUMO-specific protease are deconjugation enzymes for Ub and SUMO, respectively. Both are positively and negatively involved in cancer progression by regulating the stability of cancer progression-related proteins. Thus, inhibition of these deconjugation enzymes could provide a cancer therapeutic strategy (D’Arcy *et al.*, 2015; Kim and Baek, 2009). If UBL3 has deconjugation enzymes similar to Ub and SUMO, their inhibition will promote UBL3 modification of malignant transformation factors or growth factors, discharging them via sEVs.

Further studies to verify the correlation between UBL3 and cancer progression are necessary for initiating the development of UBL3-targeting cancer therapies.

### 3. Relationship between UBL3 and neurodegenerative diseases

sEV-related neurodegenerative diseases include AD, amyotrophic lateral sclerosis (ALS), Parkinson’s disease (PD) and Creutzfeldt-Jakob disease (Coleman *et al.*, 2012; Emmanouilidou *et al.*, 2010; Feiler *et al.*, 2015; Kunadt *et al.*, 2015; Rajendran *et al.*, 2006). AD and ALS are reported to involve etiological molecules propagated via sEVs, and these molecules are annotated as UBL3-interacting proteins (Ageta *et al.*, 2018).

AD patients develop progressive loss of memory and cognition, attributed to neurodegeneration. One etiology of AD is the extracellular accumulation of Aβ peptides that form amyloid plaques. Aβ is produced by amyloid precursor protein processing by β- and γ-secretases at MVBs, then Aβ is released into the extracellular space via sEVs to form extracellular amyloid plaques (Rajendran *et al.*, 2006).

ALS causes progressive paresis of voluntary innervated muscles due to the deterioration of cortical and spinal motoneurons, leading to death caused by respiratory failure. The aggregated transactive response DNA-binding protein 43 kD (TDP-43) is a major pathological hallmark of ALS. Although TDP-43 is localized in the nucleus under physiological conditions, mutated TDP-43 translocates to the cytoplasm, forming Ub-positive insoluble aggregates that affect motoneurons due to their toxicity. TDP-43 spreads across synaptic terminals via sEVs (Feiler *et al.*, 2015).

The neurodegenerative disease-related molecules, Aβ and TDP-43, are annotated as UBL3-interacting proteins. Thus, UBL3 is a potential therapeutic target for AD and ALS.

### 4. The isoleucine-centered hydrophobic patch as a possible UBL3-regulatory point

The development of UBL3-targeting therapies against sEV-related diseases may focus on the CAAX sequence and isoleucine-centered hydrophobic patch to regulate UBL3 activity. The CAAX sequence plays a role in substrate protein modification, as noted previously, whereas the function of the hydrophobic patch remains largely unknown. However, the isoleucine-centered hydrophobic patch is broadly conserved in Ub and UBL family members and is involved in substrate protein modification processes (Hurley *et al.*, 2006; Winget and Mayor, 2010; Zientara-Rytter and Subramani, 2019). Insights into the physiological role of the isoleucine-centered hydrophobic patch of UBL3 can be obtained from past research on Ub and UBLs, allowing discussion of its possible role as a UBL3-regulatory point.

Several UBDs recognize the isoleucine44-centered hydrophobic patch on the β-grasp of Ub via non-covalent interaction in the ubiquitin-proteasome system and during autophagy. More than twenty distinct UBD families have been identified. They include ubiquitin enzyme variants (E1s and E2s) and proteins participating in various ubiquitin signals such as protein degradation, DNA repair, and endocytosis (Hurley *et al.*, 2006; Winget and Mayor, 2010; Zientara-Rytter and Subramani, 2019). Similarly, UBD-harboring proteins that recognize the hydrophobic patch of UBLs diversify the functions of UBL families, including cellular housekeeping, nutrient recycling, chromatin remodeling, transcriptional regulation, and nucleic acid metabolism, by determining the fate of the substrates (Vierstra, 2012). Thus, UBDs diversify the fate of substrate proteins, and the isoleucine-centered hydrophobic patch is a crucial regulatory point of Ub and UBLs. For instance, APPBP1 (amyloid precursor protein-binding protein 1)-UBA3 (ubiquitin like modifier activating enzyme 3), which is the heterodimeric E1 enzyme dedicated to NEDD8, binds to the hydrophobic patch on the Ub-fold of NEDD8 non-covalently to promote thioester bond formation between its catalytic cysteine and a glycine residue in NEDD8 (Walden *et al.*, 2003).

This isoleucine-centered hydrophobic patch is also conserved in UBL3, as noted above (de la Cruz *et al.*, 2007). Thus, it is reasonable to anticipate that UBL3-regulatory factors may interact with this site. Whether UBL3 obeys the three-enzyme cascade (E1, E2, and E3) in PTM is currently unknown. If UBL3 has dedicated E1, E2 and E3 enzymes like other UBLs that mediate covalent bonding with substrate proteins, these enzymes may interact with the hydrophobic patch of UBL3. Screening hydrophobic patch-interacting molecules using *in vitro* reconstruction experiments could identify UBL3-regulatory factors and elucidate the dedicated enzyme cascade for UBL3 in the future.

### 5. UBL3 as a possible cargo-loading modality for drug delivery systems

sEVs show promise as novel DDS in an increasing number of reports (Antimisiaris *et al.*, 2018; Johnsen *et al.*, 2014). sEVs have several advantages for application to DDS compared to other nanoparticulate DDS such as liposomes: sEVs provide very high organotropism owing to their membrane proteins such as integrins and are immune-compatible because their composition is similar to that of their parent cells (Antimisiaris *et al.*, 2018).

Drug loading methodologies for sEVs can be classified into the pre-loading and post-loading methods. The pre-loading method is suitable for loading oligonucleotides or proteins and is achieved by parental cell engineering. Parental cells are treated with cargo drugs or transfected to overexpress the objective cargo proteins. Then, their cargo is packaged into sEVs prior to their release from the parental cells. For example, Mizrak *et al.* established genetically engineered sEVs by overexpressing suicide gene mRNA and protein-cytosine deaminase fused to uracil phosphoribosyltransferase in sEV-donor cells. Injection of these engineered sEVs caused schwannoma regression in a mouse model (Mizrak *et al.*, 2013). Aspe *et al.* induced apoptosis in pancreatic cancer cell lines by treating with sEVs derived from a survivin-T34A overexpressed melanoma cell line (Aspe *et al.*, 2014). In contrast, in post-loading methods, cargo drugs are loaded in sEVs after their extraction using methods conventionally used for liposomes (electroporation, simple incubation, sonication, extrusion, freeze-thaw cycles and saponin permeabilization) (Antimisiaris *et al.*, 2018). To date, this approach has mainly been limited to small molecules such as lipophilic molecules or anticancer drugs, and the loading efficiency has recently been improved. For example, Haney *et al.* (Haney *et al.*, 2015) loaded antioxidant protein catalase into sEVs using sonication and extrusion techniques. They demonstrated that the catalase was delivered across the blood-brain barrier to improve disease conditions of a PD mouse model.

Although some *in vitro* and animal experiments with protein-loaded sEVs showed therapeutic effects, most clinical research has not demonstrated therapeutic efficacy due to several disadvantages of sEV for DDS. One primary reason is the difficulty of loading sufficient drugs using either the pre-loading or post-loading method (Antimisiaris *et al.*, 2018). However, Ageta *et al.* reported that UBL3-tagged green fluorescent protein (GFP) was sorted into sEVs with higher efficiency than either Ub or SUMO-tagged GFP (Ageta *et al.*, 2018) and proposed that UBL3-tagging is a promising pre-loading strategy for generating DDS with highly efficient protein cargo loading (Ageta and Tsuchida, 2019). Further developments towards high-performance DDS using the overexpression of UBL3-tagged cargo proteins are anticipated.

## Conclusions and perspectives

The innovative concept of UBL3 that contributes to protein sorting to sEVs ranks UBL3 with the prominent Ub-proteasome and autophagy protein sorting systems. We proposed a protein sorting model based on past studies of UBL3 and other CAAX proteins. The detailed mechanism of this protein sorting process may involve cross-talk with other known protein sorting mechanisms on sEVs.

Amino acid sequence comparison of UBL3 orthologs in organisms at various stages of evolution shows that the CAAX sequence and its upstream cysteine residue are evolutionarily conserved. Furthermore, we found potential UBL3 orthologs in unicellular green algae using a BLAST search, indicating a possible new physiological role for UBL3, namely, that it is involved in not only cell-to-cell communication but also in inter-individual communications between unicellular organisms. Whether the physiological role of UBL3 in sEV protein sorting is conserved in other organisms remains to be determined.

UBL3 interacting molecules include various proteins involved in cancer progression and neurodegenerative diseases. Developing methodologies for regulating UBL3 activity may enable UBL3 as a new therapeutic target for these sEV-related diseases. In addition, we discussed the application of UBL3 as a loading modality for DDS with high loading efficiency. Versatile approaches to develop UBL3 into a therapeutic target and novel therapeutic agents are expected in the future.

## Conflict of interest

The authors declare that they have no conflicts of interest with the contents of this article.

## Author contributions

MS, TK, and KF conceived and supervised the review; YT and SK wrote the manuscript; HZ, BC, YP, KM, AK, KK, NS and MS made manuscript revisions.

## Abbreviations

A: Alanine
AD: Alzheimer’s disease
ALS: Amyotrophic lateral sclerosis
An: *Aspergillus nidulans*
At: *Arabidopsis thaliana*
ATG: Autophagy-related protein
Aβ: β-amyloid
C: Cysteine
Ci: *Ciona intestinalis*
Cn: *Cryptococcus neoformans*
Cr: *Chlamydomonas reinhardtii*
Cs: *Chlorella sorokiniana*
DDS: Drug delivery system
Dm: *Drosophila melanogaster*
Dr: *Danio rerio*
EMT: Epithelial to mesenchymal transition
ESCRT: Endosomal sorting complex required for transport
FT: Farnesyltransferase
GFP: Green fluorescent protein
hnRNP2B1: Heterogeneous nuclear ribonucleoprotein A2B1
Hs: *Homo sapiens*
HSP90: Heat shock protein 90
I: Isoleucine
IMCT: Isoprenylcysteine carboxyltransferase
Ki: *Kockovaella imperatae*
KO: Knock out
L: Leucine
Mm: *Mus musculus*
MMP: Matrix metalloprotease
Mn: *Monoraphidium neglectum*
MUB: Membrane-anchored ubiquitin-fold protein
MVB: Multivesicular body
NEDD8: Neuronal precursor cell-expressed, developmentally down-regulated 8
Nv: *Nematostella vectensis*
Os: *Oryza sativa*
PD: Parkinson’s disease
PGGTI: Geranylgeranyltransferase type 1
PTM: Post-translational modification
Py: *Patinopecten yessoensis*
sEV: Small extracellular vesicle
Sm: *Stegodyphus mimosarum*
SUMO: Small ubiquitin-like modifier
TDP-43: Transactive response DNA-binding protein 43
Ts: *Tetrabaena socialis*
Ub: Ubiquitin
UBD: Ubiquitin-binding domain
UBL: Ubiquitin-like protein
UBL3: Ubiquitin-like 3
UFM1: Ubiquitin-fold modifier 1
V: Valine
Xt: *Xenopus tropicalis*
Yl: *Yarrowia lipolytica*
